# Trends in the Discovery of New Marine Natural Products from Invertebrates over the Last Two Decades – Where and What Are We Bioprospecting?

**DOI:** 10.1371/journal.pone.0030580

**Published:** 2012-01-20

**Authors:** Miguel Costa Leal, João Puga, João Serôdio, Newton C. M. Gomes, Ricardo Calado

**Affiliations:** 1 Department of Biology and CESAM, University of Aveiro, Aveiro, Portugal; 2 Skidaway Institute of Oceanography, Savannah, Georgia, United States of America; 3 Bio3 – Estudos e Projectos em Biologia e Valorização de Recursos Naturais, lda., Almada, Portugal; Heriot-Watt University, United Kingdom

## Abstract

It is acknowledged that marine invertebrates produce bioactive natural products that may be useful for developing new drugs. By exploring untapped geographical sources and/or novel groups of organisms one can maximize the search for new marine drugs to treat human diseases. The goal of this paper is to analyse the trends associated with the discovery of new marine natural products from invertebrates (NMNPI) over the last two decades. The analysis considers different taxonomical levels and geographical approaches of bioprospected species. Additionally, this research is also directed to provide new insights into less bioprospected taxa and world regions. In order to gather the information available on NMNPI, the yearly-published reviews of Marine Natural Products covering 1990–2009 were surveyed. Information on source organisms, specifically taxonomical information and collection sites, was assembled together with additional geographical information collected from the articles originally describing the new natural product. Almost 10000 NMNPI were discovered since 1990, with a pronounced increase between decades. Porifera and Cnidaria were the two dominant sources of NMNPI worldwide. The exception was polar regions where Echinodermata dominated. The majority of species that yielded the new natural products belong to only one class of each Porifera and Cnidaria phyla (Demospongiae and Anthozoa, respectively). Increased bioprospecting efforts were observed in the Pacific Ocean, particularly in Asian countries that are associated with the Japan Biodiversity Hotspot and the Kuroshio Current. Although results show comparably less NMNPI from polar regions, the number of new natural products per species is similar to that recorded for other regions. The present study provides information to future bioprospecting efforts addressing previously unexplored taxonomic groups and/or regions. We also highlight how marine invertebrates, which in some cases have no commercial value, may become highly valuable in the ongoing search for new drugs from the sea.

## Introduction

Oceans, due to the area they represent and the ecosystem services they provide, are fundamental to our planet. They also harbour a huge biodiversity of life. Of all animal phyla described so far, only phylum Onychophora is not recorded in marine waters, while 15 phyla occur exclusively in the world's oceans [1]. Invertebrates comprise approximately 60% of all marine animal diversity [2]. Most of them belong to phyla Annelida, Arthropoda, Bryozoa, Cnidaria, Echinodermata, Mollusca, Platyhelminthes, Porifera and sub-phylum Tunicata. Although tunicates belong to phylum Chordata, several studies addressing marine invertebrates also include this group of organisms [3], [4]. Many marine invertebrates are sessile and soft bodied, and therefore must rely on chemical defences (also known as natural products), which arose through their evolutionary history to deter predators, to keep competitors away or to paralyze prey [5], [6].

The abundance and diversity of natural products (NP) having biological activity leads to an opportunity for the discovery of drugs [6]. Despite its relatively recent advent [7], [8], the bioprospecting of new marine natural products (NMNP) has already yielded several thousand novel molecules. Moreover, given that the ocean's biodiversity is higher than that recorded on land, it is expected that the discovery of NMNP will increase in the years to come, providing new and improved therapeutics for human illnesses, along with other innovative products for other industrial activities (e.g. nutraceutics and biotechnology) [9]–[11]. In order to survey chemical diversity in an efficient and effective way, one is required to employ optimized sampling strategies. Three different sampling strategies are commonly used [12]: (i) exploring untapped geographical sources; (ii) exploring new groups of marine organisms; or (iii) combining both of these sampling strategies. Geographical selection of collection sites is a highly relevant aspect in bioprospecting efforts, as it is the first step for discovering NMNP [6]. In addition, collection sites must be carefully chosen, in order to offer a combination of high biological diversity and density, such that it maximizes the number of different species being sampled and avoid adverse impacts to the collection site. Impact assessment of the sampling site is also a major concern that is essential when monitoring chemical diversity, as the loss of biodiversity through over-exploitation and habitat degradation are currently primary issues in marine conservation [13]. To our knowledge, the geographical sources of NMNP have not been thoroughly analysed and no trends for collections based upon geographical data have been published.

Worldwide marine regions can be organized by political borders (Economic Exclusive Zones - EEZ), ecological criteria (e.g. Large Marine Ecosystems - LME) and/or biodiversity (Biodiversity Hotspots - BH). EEZ are areas over which a state has jurisdiction concerning the exploration and use of its marine resources [14]. LME are near shore regions characterized by similar depth, hydrography, productivity, and trophically dependent populations [15]. LME attempts to map distinct ecological communities similar to those carried out for terrestrial ecosystems [16]. LME were conceived as units for the practical application of transboundary management issues [17]. BH are areas featuring incomparable concentrations of endemic species, which are currently experiencing exceptional loss of habitat [18]. BH boundaries are determined by “biological commonalities”, i.e., each BH features separate biota or communities of species that fit together as a biogeographic unit. Most available reviews addressing the discovery of NMNP have ignored detailed geographic information and have only briefly focused on the taxonomic position of source organisms [19]–[23]. The majority of these reviews do not consider taxonomical levels lower than phylum and mainly analyse the number of NMNP and their chemical properties.

The evaluation of geographical and taxonomical trends on NMNP collection sites can provide important information for future bioprospecting efforts worldwide. The goal of this study is to analyse the trend of NMNP discovery over the past two decades, taking into consideration different taxonomical levels and geographical approaches. The questions addressed in this study were: 1) what have been the main invertebrate taxa that provided most NMNP? 2) where have those taxa been collected? and 3) what was the trend of collected taxa and explored regions over the last two decades?. Beyond the importance of the outcome of the present work for several research fields associated with marine natural products (MNP) (e.g. chemical ecology, biotechnology and aquaculture), this study is also intended to alert nations worldwide regarding the value of marine biodiversity. The Oceans have been shown to be a last stronghold of global biodiversity, one often not properly valued by human society. By recognizing the importance of marine biodiversity to bioprospecting, developing nations may find extra reasons to advocate marine conservation.

## Methods

In order to gather available information on NMNPI, the yearly reviews of Marine Natural Products published by Natural Product Reports were surveyed [19]–[38]. Information for the years 1990 to 1999 and 2000 to 2009 was assembled. Information on source organisms, particularly taxonomical information and collection sites, was assembled along with the NP discovered. When particular information was insufficient or omitted, the original article describing the discovery of the NP was consulted in order to retrieve data that was as accurate as possible. Note that it was not always possible to retrieve all missing information by consulting the original article, as some of those works were written in languages other than English while others provided no detailed information about the sampling site. About 8% of all NMNPI recorded in the present study lacked sufficient information for one of these criteria.

The World Register of Marine Species (WoRMS) database was used to provide detailed taxonomical information (phylum, subphylum, class, subclass, order, family and genus) for each surveyed species and to validate and/or update their scientific names [39]. WoRMS database was also used to enumerate the total number of species belonging to distinctive marine invertebrate phyla, subphyla, classes, subclasses and orders currently recognised as valid. As previously mentioned, although several studies addressing NP from marine invertebrates commonly include tunicates, this group of organisms belong to phylum Chordata [3], [4]. In this way, NMNPI isolated from tunicates were also considered in the present work, and thus every time that Chordata is mentioned throughout the text it refers exclusively to tunicates.

Information on the collection site of each source organism was used to identify each NP. This information was employed to determine the following geographical categories: country, continent, ocean, latitude, EEZ (list available at www.seaaroundus.org/eez/), LME (list available at http://www.lme.noaa.gov/) and BH (list available at www.biodiversityhotspots.org). Six continents (Africa, America, Antarctica, Asia, Europe, Oceania) and five oceans (Antarctic, Arctic, Atlantic, Indian, Pacific) were defined. Latitude was organized in polar (above the Arctic Circle and below the Antarctic Circle), temperate (between the Tropic of Cancer and the Arctic Circle and between the Tropic of Capricorn and the Antarctic Circle) and tropical, and each was divided in North and South (between the Tropic of Cancer and Tropic of Capricorn). Concerning EEZ, the data of external territories, such as provinces, overseas departments, etc., were separated from their parent country. The information regarding those external territories was treated as a separate EEZ. The geographical information was mapped using Manifold® 8.0 software.

Changes on NMNPI over decades were analysed through percentage decreases or increases. To calculate percentage changes we first subtracted the sum of new NP for the 2000s from the sum of the NP for the 1990s. Afterwards, the difference obtained was divided by the NP value for the 1990s and multiplied by 100. The total number of species yielding new NP, the average number of marine invertebrate species yielding new NP (calculated as the percentage of the number of species with new NP divided by the total number of valid species in that particular taxonomic group; e.g., taxon X has 100 species and for 50 of them new NP were discovered, therefore 50% of the species of that taxon have new NP), and the average number of NMNP per invertebrate species with NP (calculated by dividing the total number of NP of a particular taxonomic group by the total number of species of that group for which new NP were reported; e.g. 200 new NP were discovered in 50 species from taxon X, therefore, in average, taxon X has 4 new NP.species^−1^), were also determined for the taxonomic levels and geographic categories detailed above. As the present study analysed all NMNPI discovered over the 1990s and 2000s, no statistical analyses were conducted to determine surveyed trends over these two decades.

## Results

The present work covered a total of 9812 NMNPI discovered from 1990 to 2009. A difference of +17.7% on the number of NMNPI discovered over the two decades was recorded, with an average (± standard deviation) of 450.8±70.9 and 530.4±63.2 NMNPI discovered over each year in the 1990s and the 2000s, respectively.

### Taxonomical trends

A total of 11 phyla, 6 subphyla, 20 classes, 20 subclasses, 74 orders, 253 families, 569 genera and 1354 species encompassed the recorded NMNPI. Phylum Porifera comprised 48.8% of all NMNPI discovered since 1990, while Cnidaria comprised 28.6%. Other noteworthy phyla were the Echinodermata, Chordata and Mollusca, which represented 8.2%, 6.9% and 5.8%, respectively. The remaining 1.7% were covered by the following phyla in decreasing order of importance: Annelida, Bryozoa, Platyhelmintes, Hemichordata, Brachiopoda and Arthropoda. Only Porifera (+8.9%; from 2291 to 2496 new NP) and Cnidaria (+72.0%; from 1031 to 1773 new NP) recorded a noteworthy increase in the number of NMNP discovered between decades.

#### Trends for Porifera and Cnidaria

**Figure 1:**
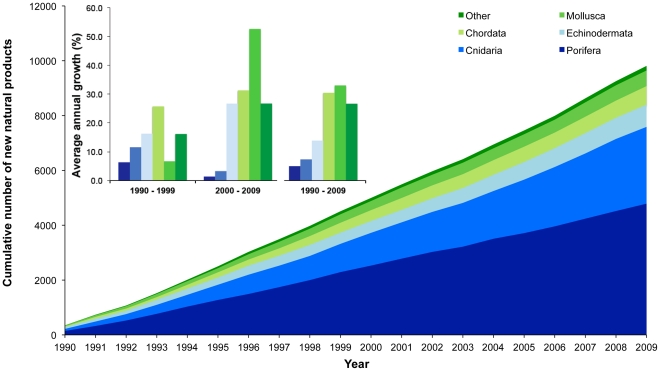
New natural products from marine invertebrate phyla. Cumulative number of new natural products discovered from different marine invertebrate phyla from 1990 to 2009 (Group “Other phyla” include Annelida, Arthropoda, Brachiopoda, Hemichordata, Platyhelmintes and Bryozoa). Inset: Annual growth of the number of new marine natural products from different marine invertebrate phyla discovered in the 1990s, 2000s and during both decades.

New NP from Porifera showed a notable increase in the first 5 years of the 1990's and subsequently maintained a flat trend with an average of 250.2±29.3 NMNP.year^−1^ (1995–2009). Discovery of NMNP from Cnidaria also increased over decades and, despite some variation, it has maintained a noticeable increase in the past 20 years. Both Porifera and Cnidaria have been responsible for the discovery of most NMNPI since 1990, with the contribution of other phyla being relatively low (Figure 1). In contrast, Figure 1 also shows that although Porifera and Cnidaria have been the most important sources of NMNPI, their relative annual growth has been relatively lower when compared to that displayed by other phyla.

**Figure 2:**
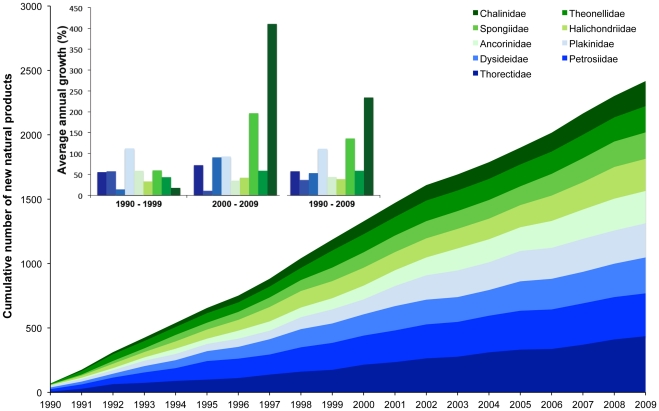
New natural products from Porifera. Cumulative number of new natural products discovered from the most representative families in phylum Porifera from 1990 to 2009. Inset: Annual growth of the number of new marine natural products discovered from the most representative families in phylum Porifera in the 1990s, 2000s and during both decades.

Concerning the 4 classes within the Porifera, Demospongiae accounted for more than 99.8% of NMNPI. Although NMNP were recorded in 17 orders of Demospongiae, about 89.4%of the NP were derived from only 8 of those orders (see Table 1). All NMNP discovered since 1990 were recorded in 64 families belonging to the Porifera. However, about 51% of these products were derived from only 9 families: Thorectidae, Petrosiidae, Dysideidae, Plakinidae, Ancorinidae, Halichondriidae, Spongiidae, Theonellidae and Chalinidae. Although all these families equally account for the overall NMNP derived from Porifera, the highest increase of the number of NMNP annually discovered was recorded for the families Chalinidae and Spongiidae (Figure 2). The genera that provided most NMNP were *Plakortis*, *Dysidea*, *Haliclona* and *Petrosia*, each one accounting for approximately 4% of all Porifera NMNP. While increases were observed for *Plakortis* (34.8%; from 92 to 124 NP) and *Haliclona* (+48.6%; from 74 to 110 NP), noteworthy decreases were observed for *Dysidea* (−23.6%; from 106 to 81) and *Petrosia* (−34.6%; from 104 to 68 NP) as well as for *Xestospongia* (−71.4%; from 105 to 30 NP), *Theonella* (−65.1%; from 106 to 37 NP) and *Pseudoceratina* (−46.8%; from 77 to 41 NP).

**Table 1:** Number of new natural products (NP) discovered in the most representative taxa of phylum Cnidaria and Porifera in the last two decades.

Taxon	New NP in the 1990s	New NP in the 2000s	Decade variation of new NP (%)
**Phylum Cnidaria**	1031	1773	+72.0
Class Anthozoa	1017	1758	+72.9
Sub-class Octocorallia	963	1715	+78.1
Order Alcyonacea	934	1694	+83.6
Family Alcyoniidae	293	489	+66.9
Family Briareidae	158	156	−1.3
Family Clavulariidae	41	150	+265.9
Family Gorgoniidae	109	165	+51.4
Family Nephtheidae	58	227	+291.4
Family Plexauridae	97	99	+2.1
Family Xeniidae	72	147	+107.0
**Phylum Porifera**	2291	2496	+8.9
Class Demospongidae	2287	2492	+9.0
Order Astrophorida	126	227	+80.2
Family Ancorinidae	86	165	+91.9
Order Dictyoceratida	488	610	+25.0
Family Dysideidae	151	128	−15.2
Family Thorectidae	175	261	+49.1
Family Petrosiidae	209	124	−40.7
Order Halichondrida	321	286	−10.9
Family Halichondriidae	133	117	−12.0
Order Haplosclerida	424	403	−5.0
Order Homosclerophorida	110	155	+40.9
Family Plakinidae	110	155	+40.9
Order Lithistida	166	91	−45.2
Order Poecilosclerida	264	334	+26.5
Order Verongida	143	122	−14.7

Footnote: Only the classes, sub-classes, orders and families that accounted for, at least, 5% of the new natural products discovered for the respective phylum are presented.

**Figure 3:**
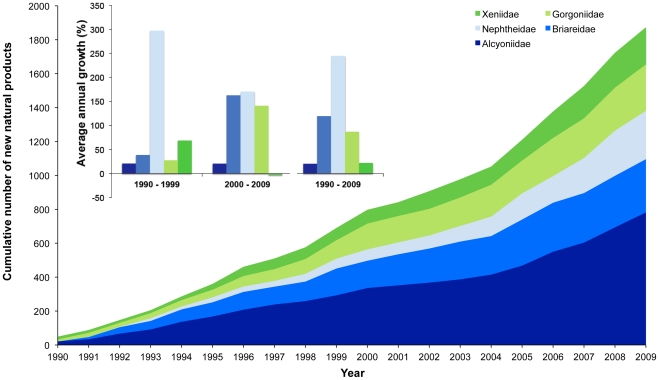
New natural products from Cnidaria. Cumulative number of new natural products discovered from the most representative families in phylum Cnidaria from 1990 to 2009. Inset: Annual growth of the number of new marine natural products discovered from the most representative families in phylum Cnidaria in the 1990s, 2000s and during both decades.

The class Anthozoa comprised 99.0% of NMNP recorded from Cnidaria. Discovery of new NP from class Anthozoa increased 72.0% from the 1990s to the 2000s (Table 1). The sub-class Octocorallia accounted for the most NMNP in the Anthozoa (95.5%), order Alcyonacea, accounted for 98.1% of new NP from Octocorallia and 26.8% of all NMNPI. Alcyonacea had a notable increase between decades (Table 1), which was explained by the last 5 years of the current survey, when an average of 207.4±27.6 new NMNP.year^−1^ was recorded (from 1990 to 2004 the average was 106.1±28.7 NMNP.year^−1^). Although NMNP were recorded for 19 Alcyonacea families, 71% of these belonged to only 5 families (see Figure 3). Alcyoniidae encompassed most of the NMNP from Cnidaria, and covers 8.0% of all NMNPI discovered since 1990. In regard to genus level, all dominant genera belonged to the alcyonacean families represented in Figure 3. The main genera were *Sinularia* (11.9%), *Briareum* (11.2%), *Pseudopterogorgia* (6.6%), *Sarcophyton* (6.1%) and *Nephthea* (5.2%). The genera that recorded most noteworthy changes between decades were *Nephthea* (+468.2%; from 22 to 125 NP), *Clavularia* (+255.2%; from 29 to 103 NP), *Junceella* (+229.2%; from 24 to 79 NP) and *Sarcophyton* (+89.8%; 59 to 112 NP). The cnidarian species that accounted for the highest number of NMNP since 1990 were *Clavularia viridis* (23 publications described a total of 96 NP), *Pseudopterogorgia elisabethae* (24 publications described a total of 86 NP) and *Briareum excavatum* (56 NP), and each accounted for approximately 3% of all cnidarian's NMNP. Using the example of these 3 species, an average of 3.6 to 5 new NP were reported per publication.

#### Trends for other phyla

Most of the echinoderms' NMNP were from sub-phylum Asterozoa (54.9%), while sub-phylum Echinozoa accounted for 33.7%. Together, the classes Asteroidea (529 NP) and Holothuroidea (213 NP) accounted for 91.7% of Echinodermata NMNP. The order Valvatida (257 NP) accounted for 31.8% of NMNP from Echinodermata (31.8%). No noticeable trends over decades were observed for phyla or other lower Echinodermata taxonomic levels. Concerning sub-phylum Tunicata, all NMNP were from class Ascidiacea, from which 83.0% belong to order Aplousobranchia. Aplousobranchia families yielding the highest numbers of NMNP were Didemnidae (33.6%; 240 NP) and Polyclinidae (16.5%; 112 NP). The most represented genera were *Aplidium* (84 NP), *Didemnum* (90 NP) and *Lissoclinum* (94 NP). In regard to phylum Mollusca, of the 8 classes currently recognised, only classes Bivalvia and Gastropoda were represented, with the latter clearly dominating (82.7% of all Mollusca's NMNP; 472 of the 571 NP). While the number of NMNP from the Gastropoda decreased from 279 to 193 NP over decades, the number of NMNP from Bivalvia increased 109.4% (from 29 to 70 NP). Order Nudibranchia accounted for 169 of all NMNP discovered from Gastropoda since 1990, although it decreased 72.3% between decades (from 116 to 53 NP). *Aplysia* should be distinguished from all molluscs as it yielded 58 new NP (10.2% of mollusc's new NP). Although this genus holds over 40 valid species, a single species (*Aplysia dactylomela*) was responsible for the discovery of 26 new NP.

#### Bioprospecting efforts and natural products richness

According to the WoRMS database, the 11 phyla recorded in the present study currently display about 170000 valid species [39]. However, since 1990, most research on NMNPI has been focused on less than 1% of the biodiversity currently recognised for marine invertebrates. When assessing the most bioprospected taxa for NMNP since 1990, 7.4% of all Porifera species have yielded NMNP, while for phyla Cnidaria and Echinodermata only 2.8% and 2.1% of their species yielded NMNP, respectively. For all other phyla, NMNP were detected in less than 1% of their species with the exception being the sub-phylum Tunicata, as 4.3% of its species yielded NMNP.

The average number of NMNP per species, for which new NP have been documented since 1990, showed that Cnidaria (9 NMNP.species^−1^), Hemichordata (9 NMNP.species^−1^) and Porifera (8 NMNP.species^−1^) possessed the highest numbers of NMNP per species. Other noteworthy phyla, such as Bryozoa, Chordata (tunicates) and Echinodermata, recorded approximately 5 NMNP.species^−1^. Table 2 summarizes the most important invertebrate orders yielding NMNP, which accounted for 90% of all NMNPI discovered since 1990. The most bioprospected orders containing NMNP (i.e., with high number of species with NMNP) belonged to sponges, particularly Verongida, Agelasida, Dendroceratida, Dictyoceratida and Homosclerophorida. However, apart from Dictyoceratida, all other orders had relatively low species richness. Although not displayed in Table 2, the orders Helioporacea (Cnidaria, sub-class Octocorallia) and Cyrtocrinida (Echinodermata, class Crinoidea) had 25% of their taxa yielding NMNP. However, these taxa only accommodate 4 described species. Five orders registered an average ≥10 NMNP.species^−1^ (Table 2). Among these orders, Alcyonacea and Dictyoceratida are noteworthy because of the high number of species bioprospected since 1990.

**Table 2:** Number of species with new natural products (NP) and other ratios regarding species richness.

Taxon	Number of valid species	Number (%) of species with new NP	Average number of new NP per species 2
**Phylum Chordata (sub-phylum Tunicata)**	2959	128 (4.3)	5.3
Class Ascidiacea	2814	120 (4.3)	5.7
Order Aplousobranchia	1480	96 (6.5)	5.9
**Phylum Cnidaria**	10923	302 (2.8)	9.3
Class Anthozoa	7041	300 (4.3)	9.3
Order Alcyonacea	3243	257 (7.9)	10.2
**Phylum Echinodermata**	7353	153 (2.1)	5.3
**(sub-phylum Asterozoa)**	4018	72 (1.8)	6.2
Class Asteroidea	1849	74 (4.0)	7.2
Order Forcipulatida	273	22 (8.1)	5.2
Order Spinulosida	131	7 (5.3)	12.4
Order Valvatida	733	25 (3.4)	10.1
**(sub-phylum Echinozoa)**	2890	58 (2.0)	0.3
Class Holothuroidea	1800	51 (2.8)	4.2
Order Dendrochirotida	794	25 (3.3)	5.2
**Phylum Mollusca**	35407	147 (0.4)	3.9
Class Gastropoda	24655	125 (0.5)	3.8
Order Anaspidea	44	14 (31.8)	6.6
Order Nudibranchia	1673	47 (2.8)	3.6
**Phylum Porifera**	8030	593 (7.4)	8.1
Class Demospongiae	6780	601 (8.9)	8.0
Order Agelasida	41	17 (41.5)	8.3
Order Astrophorida	675	55 (8.2)	6.4
Order Dendroceratida	70	16 (22.9)	6.0
Order Dictyoceratida	465	106 (22.8)	10.4
Order Hadromerida	747	30 (4.0)	4.5
Order Halichondrida	675	76 (11.3)	8.0
Order Haplosclerida	1060	96 (9.1)	8.6
Order Homosclerophorida	83	16 (19.3)	16.6
Order Lithistida	197	27 (13.7)	9.5
Order Poecilosclerida	2482	102 (4.1)	5.9
Order Verongida	79	33 (41.8)	8.0

Footnote: Only the orders that account for 90% of total New Natural Products from Marine Invertebrates discovered since 1990 are presented, as well as their phylum and class. Species richness information gathered from WoRMS database.

1 The percentages of species of particular taxa for which new NP were discovered since 1990.

2 The average number of new NP per species of particular taxa derived from the species where new NP were reported since 1990.

### Geographical trends

It was not possible to assign all NMNPI identified in the present work to a given geographical area. However, unmatched compounds accounted for only 4% of all NMNPI, except for the categories BH (6.9%) and LME (5.9%). For all EEZ, BH and LME regions no trend was found between the number of NMNPI and the area covered by each region.

#### Latitudinal trends

The northern hemisphere accounted for 62.6% (6145 NP) of all NMNPI discovered since 1990, whereas the tropics accounted for 55.1% (5403 NP). The number of NMNPI decreased in the 2000s for both temperate regions. In the temperate North a decrease of 6.7% was recorded over decades (less 1707 NP were discovered in the 2000s), while in the temperate South a 32.4% decrease was observed (less 386 NP discovered in the 2000s). In contrast, an increase of 18.8% (from 1290 to 1533 NP) and 103.9% (from 649 to 1731 NP) was observed in the North and South tropical regions, respectively. Porifera and Cnidaria were the dominant sources of NMNPI from temperate (Porifera 46.9%, Cnidaria 19.6%) and tropical regions (Porifera 52.1%, Cnidaria 35.4%). However, in polar regions, Echinodermata was the dominant source of NMNPI (40.2%; 80 of the 199 NP). For all latitudinal regions, the order Alcyonacea has been the main source of NMNPI since 1990 (polar 19.6%, temperate 16.5%, tropical 34.6%), followed by Dictyoceratida and Haplosclerida, each accounted for 8 to 13% at both temperate and tropical regions, respectively. For polar regions, specifically the south pole, Spinulosida (phylum Echinodermata, class Asteroidea) was the second dominant order, having 16.1% of all the 177 NMNPI discovered since 1990. The most noteworthy genus was *Sinularia*, which accounted for 5.4% of NMNPI discovered in tropical regions since 1990. Regarding the number of species yielding NMNP, the highest numbers were registered in the northern tropical and temperate regions as each recorded ∼38% of the total number of species registered in the present study. Nonetheless, the highest number of NMNPI per species was observed in the southern tropical and northern temperate regions (∼6 NP.species^−1^), while the lowest records were discovered for the southern polar and southern temperate regions (∼4 NP.species^−1^).

#### Oceans and Continents trends

Most of the NMNPI were discovered in the Pacific Ocean (63.4%), while the Atlantic and Indian Oceans, accounted for 19.6% and 12.8%, respectively. For the Pacific Ocean a noteworthy increase of 42.6% (from 2564 to 3657 NP) was recorded. Apart from the Arctic and the Antarctic, where Echinodermata and Cnidaria were the most common source of NMNPI, the dominant sources of NMNPI in the other Oceans were phyla Porifera (44–52%) and Cnidaria (28–31%). The order Alcyonacea was the main source of NMNPI for the Pacific, Atlantic and Indian oceans, with 27 to 29% of all NMNPI discovered in each. Although NMNP from order Aspidochirotida (phylum Echinodermata) only represented 1.0% of all NMNPI discovered in the Pacific Ocean, a remarkable increase was observed between decades (+1020.0%; from 5 to 52 NP). The nudibranch genus *Doris* represented 8.5% of all Antarctic NMNPI discovered since 1990, while the gorgonian *Pseudopterogorgia* was the genus with the most NMNP in the Atlantic Ocean (7.3%). The soft coral *Sinularia* accounted for 7.9% and 3.6% of all NMNPI discovered in the Indian and Pacific Oceans, respectively. The highest number of NMNP per number species followed the same trend as the number of NMNPI for each ocean (Pacific - 8 new NP.species^−1^; Atlantic – new 5 NP.species^−1^; Indian – new 4 NP.species^−1^). The only exception was the Antarctic, which recorded a very low number of species yielding NMNP (33 species) with an average of 5 NP.species^−1^.

Almost half of all NMNPI were associated with Asian countries (45.5%), although Oceania (22.3%) and America (15.8%) also accounted for a notable fraction of the total number of NMNPI discovered since 1990. Only Africa (+23.2%; from 263 to 326 NP) and Asia (+79.6%; from 1630 to 2928 NP) registered positive trends over decades. For most continents, Porifera and Cnidaria were the main sources of NMNPI since 1990. However, it is worth noting that NMNP from Porifera dominated in Oceania (72.0%) and Africa (58.6%). Furthermore, it should be emphasized that NMNP from molluscs accounted for 26.9% of European NMNPI (a total of 144 NP) since 1990, while Porifera accounted for 35.7% (191 NP).

#### Country and Exclusive Economic Zones trends

**Figure 4:**
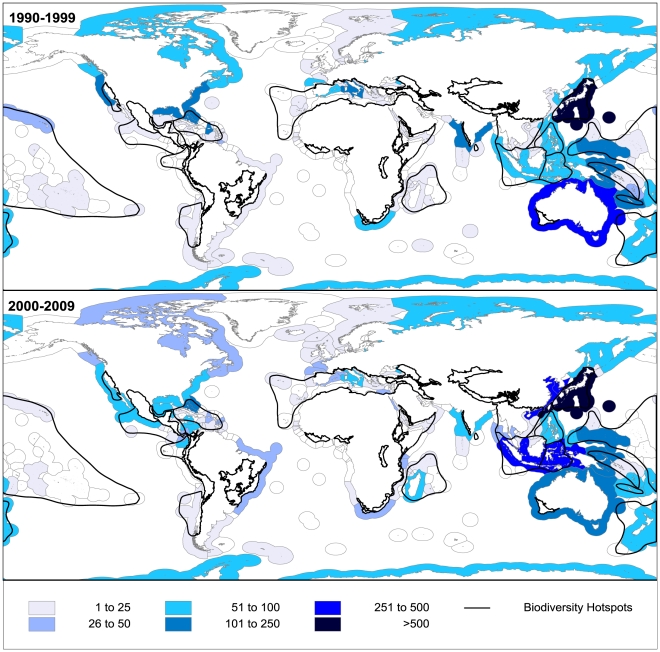
Economic Exclusive Zones. Number of new natural products from marine invertebrates for world Economic Exclusive Zones (EEZ) during the 1990s and the 2000s. Boundaries of Biodiversity Hotspots worldwide are also presented.

Since 1990, Japan clearly stands apart from all other countries, as it contained 17.3% of all NMNPI. Taiwan and Australia each represented approximately 7% of NMNPI, while the USA and China represented 5% each. Generally, the distribution of NMNPI per EEZ is very similar to the one obtained for the countries category. Altogether, the Japanese, Taiwanese, Australian, South Korean and Chinese EEZ accounted for a total of 40.4% NMNPI (Figure 4). Other important EEZ were those of Indonesia, Micronesia, Bahamas and New Caledonia (Table 3). The largest differences between countries and EEZ information were found for the United Kingdom, France and United States. While the countries United Kingdom, France and United States were associated with 39, 406 and 477 NMNPI, the NMNPI values 4, 47 and 169 were associated with the United Kingdom, France and United States EEZs. These differences are linked with overseas departments and territories, which have their own EEZs but belong to the same country. New Caledonia, Martinique and Mayotte EEZ are, for example, French territories. The highest increases over decades were recorded in the EEZ of China, Taiwan, Indonesia and South Korea, while the most noteworthy decreases were observed for New Caledonia and Puerto Rico (Table 3). When considering all NMNPI discovered since 1990, it is evident that the most bioprospected region in the world was the Indo-Pacific (Figure 4). Furthermore, Figure 4 also shows that most of the EEZs yielding the greatest number of NMNPI overlapped with worldwide BH.

**Table 3:** Number of new natural products (NP) discovered in the most important Exclusive Economic Zones in the last two decades.

Exclusive Economic Zone	New NP in the 1900s	New NP in the 2000s	Decade variation of new NP (%)
Andaman and Nicobar Islands	79	31	−60.1
Antarctica	97	80	−17.5
Australia	441	248	−43.8
Bahamas	167	125	−25.2
Canada	83	41	−50.6
China	27	397	+1370.4
Fiji	63	60	−4.8
India	147	87	−40.8
Indonesia	59	286	+384.8
Italy	114	80	−29.8
Japan	977	717	−26.6
Micronesia	211	105	−50.2
New Caledonia	234	52	−77.8
New Zealand	62	64	+3.2
Palau	83	80	−3.6
Papua New Guinea	104	124	+19.2
Philippines	83	100	+20.5
Puerto Rico	175	48	−70.6
Russia	55	87	+58.2
South Africa	99	41	−58.6
South Korea	77	350	+354.6
Spain	90	31	−65.6
Taiwan	54	684	+1166.7
United States	106	63	−40.6
Vanuatu	26	86	+230.8

Footnote: Only the Exclusive Economic Zones that accounted for, at least, 1% of the new NP discovered since 1990 are presented.

NMNP from Porifera accounted for most of NMNPI discovered in worldwide EEZ since 1990. For instance, for Vanuatu, Papua New Guinea, Bahamas, Indonesia and Australia EEZ, NMNP from Porifera accounted for 100.0%, 89.5%, 86.3%, 71.6% and 68.5% of all NMNPI discovered for each of these EEZ. In contrast, NMNP from cnidarians accounted for 95.0%, 81.2% and 48.2% of all NMNPI discovered in the EEZ of Taiwan, Puerto Rico and China, respectively. It is also worth mentioning that in the Russian EEZ, 81.0% of NMNPI were associated with Echinoderms.

#### Biodiversity Hotspots and Large Marine Ecosystems trends

**Figure 5:**
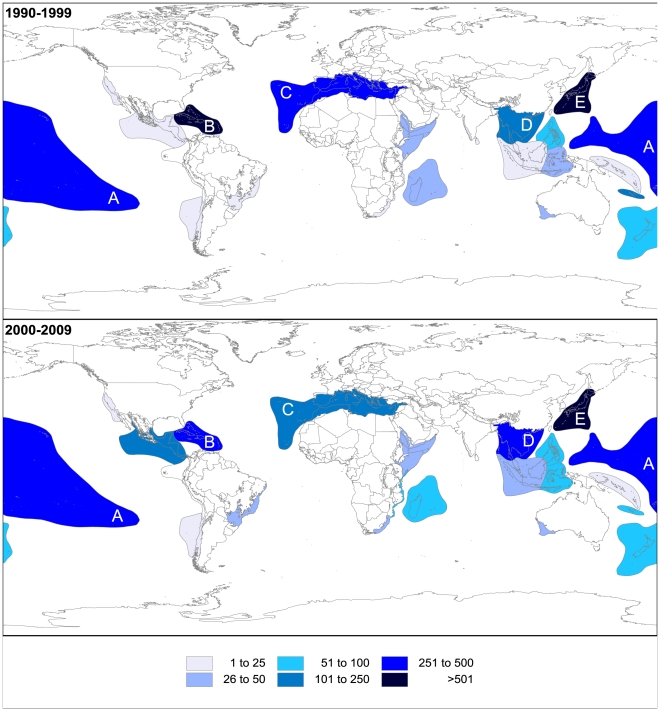
Biodiversity Hotspots. Number of new marine natural products from invertebrates (NMNPI) for Biodiversity Hotspots (BH) worldwide during the 1990s and the 2000s (A – Polynesia-Micronesia, B – Caribbean islands, C – Mediterranean basin, D – Indo-Burma, E – Japan).

Since 1990, 67.5% of all NMNPI were associated with BH, and 23 of the 34 BH recorded marine invertebrate species with NMNP (Figure 5). Overall, the BH with most NMNPI were Japan, the Caribbean Islands, Polynesia-Micronesia and Indo-Burma, with 17.3%, 9.1%, 8.3% and 5.6% of total NMNPI recorded for the analysed period, respectively. However, the biggest increases over decades were observed for Mesoamerica (+3920.0%; 5 to 201 NP), Coastal Forests of Eastern Africa (+1820.0%; 5 to 96) and Indo-Burma (337.3%; 102 to 446 NP) BH. On the other hand, the highest decrease was found for the New Caledonia BH (−77.8%; 234 to 52 NP). For all 4 BH with most NMNPI being recorded since 1990, Porifera was the dominant source of these compounds (between 52–76% of NMNPI for each BH), apart for Indo-Burma BH, where 50.4% of NMNPI were associated with cnidarians. Alcyonacea was also the order that recorded most NMNPI for Japan, Caribbean Islands and Indo-Burma BH, accounting for 20.0%, 35.2% and 50.2%, respectively. Japan, Caribbean Islands, Polynesia-Micronesia and Mesoamerica BH recorded 7 and 5 NMNP.species^−1^ for the first two and latter two BH, respectively.

**Figure 6:**
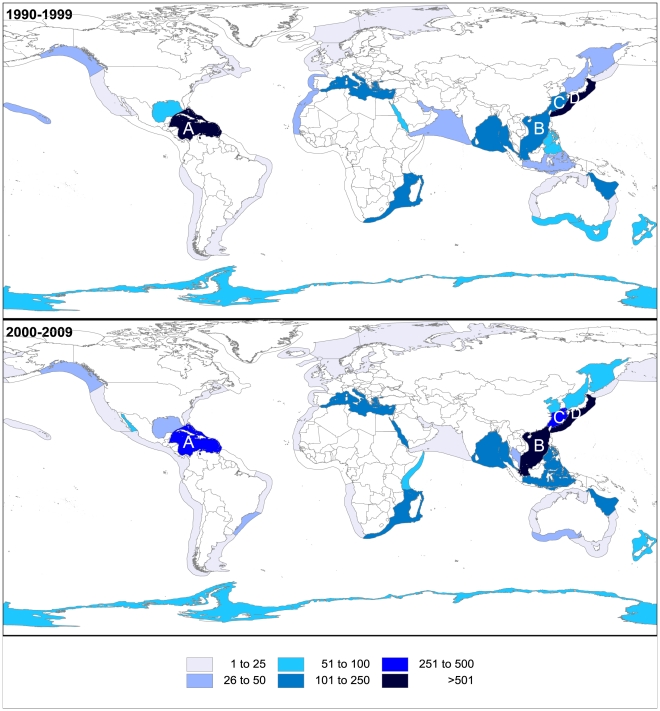
Large Marine Ecosystems. Number of new marine natural products from invertebrates (NMNPI) for world Large Marine Ecosystems (LME) during the 1990s and the 2000s (A – Caribbean Sea, B – South China Sea, C – East China Sea, D – Kuroshio Current).

The information displayed in Figure 6 shows the number of NMNPI discovered in the last two decades for LME worldwide. Overall, Kuroshio Current (15.5%), Caribbean Sea (10.0%), South China Sea (9.8%), East China Sea (5.9%), Mediterranean Sea (4.2%), Northeast Australian Shelf (3.6%) and Bay of Bengal (2.8%) accounted for more than half of all NMNPI discovered since 1990. Some of the most noteworthy increases over decades were observed for the South China Sea (+730.0%; 103 to 855 NP), Indonesian Sea (+435.3%; 34 to 182 NP) and East China Sea (+115.3%; 183 to 394 NP). Most of the decreases were registered for the LME surrounding Australia (Figure 6). Generally, Porifera and Cnidaria species dominated NMNPI on all LME. The exceptions were the Humboldt Current, where molluscs accounted for 94.4% of NMNPI, the Iberian Coastal, where molluscs were the dominant source of NMNP (43.6% - most of them from genus *Aplysia*), the Sea of Okhotsk, where the Echinodermata were the main source of NMNPI (95.1%), and the Sea of Japan, where Echinodermata accounted for 51.8% of all NMNPI.

## Discussion

This study investigated the taxonomical and geographical trends of NMNPI discovery from 1990 to 2009. If a particular species or taxonomical group does not show a NMNP, it means that no NMNP was reported associated with that species/group in the past two decades. It does not necessarily indicate that the specific species/group does not have any NP. Several excellent marine chemical ecology reviews already exist [9], [40], [41] and this paper does not attempt to provide a comprehensive overview of all NMNPI or provide unconditional statements about their discovery.

### Marine natural product research: the case of marine invertebrates

Research on marine natural products began in the 1950s [42], at a time when important breakthroughs on the taxonomy of marine animals took place [43]. This research field expanded during the 1970s and 1980s and only by the end of the 1980s and the beginning of the 1990s began to appear as an economically appealing activity [44], [45]. From 1990 to 2005, about 800 NMNP were discovered each year [43], with approximately 66% of those compounds recorded in marine invertebrates. The finding of NMNPI, since 1990, has followed the same increasing trend of NMNP. Thus between the 1990s and the 2000s a noteworthy increase was recorded for NMNPI. Between 2005 and 2009, however, the fraction of NMNP yielded by invertebrates decreased from 63.2% (2005) to 55.1% (2009). Nevertheless, marine invertebrates have continued to cover a substantial fraction of all NMNP discovered each year. This trend did not reflect the decision of pharmaceutical companies, which have been the major driving force supporting bioactive compound research until deciding to close most of their programs for the search of NMNP in the 1990s [10], [41], [46], [47]. Seemingly, the quest for NMNP have benefited from a renaissance in the last 5 years, namely due to the development of new methods in analytical technology, spectroscopy and high-throughput screening [10]. It also benefited from the failure to deliver new drug leads in meaningful numbers by competing technologies, such as chemical synthesis. This scenario possibly explains the growth of NMNPI discovery since 2005.

### Key source organisms of new natural products

As highlighted by other reviews [41], [48], phyla Porifera and Cnidaria have been the two main sources of NMNP. The NMNP discovery trends in the last two decades from these phyla show a stable tendency in the discovery of these compounds from sponges in the last decade. In contrast, the positive trend recorded for cnidarians probably shows that the bioprospecting effort on these organisms has been continuously increasing since 1990. Although several Porifera taxa harbor larger number of species, most NMNP from Porifera have been associated only with one class, Demospongiae, and a few of its orders: Dictyoceratida, Haplosclerida, Halichondrida, Poecilosclerida and Astrophorida. The same feature was observed for Cnidaria, as most NMNP were recorded from class Anthozoa and order Alcyonacea. For both Porifera and Cnidaria, only a lower fraction (<10%) of their total number of species has yielded new NP in the past two decades, with this fraction being considerably higher in Porifera. This result is probably associated with the popularity of Porifera for the bioprospecting of new NP since the beginning of MNP research. Although Porifera have been the dominant source of NMNPI over the past two decades, this trend may change as fewer unscreened species become available for study. Alternatively, researchers may find new opportunities to identify new sponge-derived NP by focusing their efforts on organisms that belong to orders with large chemical diversity and yet with a relatively low number of screened species. This trend is getting underway for the Astrophorida and Poecilosclerida, as an increase in the numbers of NMNP reported from these two orders was observed. Shifts between decades observed for Porifera taxa could be associated with social trends, as unscreened taxa become very popular, while others become obsolete due to more intense screening efforts. The shifts recorded in our study can also be linked with the increasing popularity of bioprospecting and the preference of new research groups to target new source organisms, attempting to differentiate their work from those of well-established research groups.

Although Porifera has been the major source of NMNPI, one should underline the cnidarian order Alcyonacea, particularly the family Alcyoniidae and genera *Sinularia* and *Briareum*, due to the large number of new NP discovered for these groups. Nonetheless, do these results mean that Porifera and Cnidaria are the best sources of NMNPI? Both phyla represent the highest number of NMNP per species, apart from Hemichordata, whose data results from 1 single species. However, this could be a consequence of the popularity of these organisms amongst researchers conducting bioprospecting efforts [22], once they have historically been pointed out as “easy” sources of NMNP [45], [48]. It is also important to highlight that if one study discovered 20 new NP in species X, one can expect that similar taxa may hold a great potential for finding new NP. Additionally, it is possible that the same taxa may have new NP yet to be discovered. This most likely occurs when studies are only targeting specific groups of new NP and do not describe all NP that may exist in the studied organism. While some studies describe the discovery of several new NP along with the compounds already known (e.g. [49]–[51]), other studies only report the new NP discovered (e.g. [52], [53]). If several publications, from different research groups, report new NP from a particular taxon, it is indicative of the popularity of that taxon. For instance, *Sinulara flexibilis*, a popular soft coral, has 35 new NP reported in 5 studies from different teams [49]–[51], [54]. The popularity of sponges and cnidarians may also be explained by the accessibility of biomass to researchers and the wider distribution of the most targeted species. Such abundance and distribution would allow several research groups to sample all the necessary biomass and to systematically explore the chemical compounds of target species. Moreover, these two features are also extremely important to consider when analyzing the economic feasibility of harvesting biomass for drug development.

Besides sponges and corals, results also underline other interesting sources of NMNP, such as echinoderms, tunicates and molluscs. Despite the absence of noteworthy inter-decadal changes on NMNP from echinoderms, tunicates and molluscs, some taxa within these phyla have shown to yield a relatively high number of NMNP per species. The sea star *Certonardoa semiregularis* and the sea slug *Aplysia dactylomela* exemplify this. Although this study also analyzed results at the species level, most focus was at higher taxonomic levels. This decision was based on the challenges associated with the accurate identification of several marine invertebrates to species level [55]. Indeed, the correct identification of source organisms is of major importance to avoid duplication of already known information and, more importantly, to accurately analyze the chemical diversity associated with a particular species from different regions. By analyzing the trends of higher taxonomic levels, such as genus, one can avoid the bias generated by misidentifications [55]. Nonetheless, it is important to highlight that NP diversity usually varies at the species level and the existence of undetected cryptic species complexes can mask potentially valuable resources for drug discovery [56], [57]. Another factor that probably contributes to the variation of chemical diversity at the species level is the microbial community associated with the marine invertebrate. There is growing evidence suggesting that microbes associated with marine invertebrates may be the true producers of some of the NP that were previously assumed to be produced by their invertebrate host [9], [58], [59]. The symbiotic community of microorganisms living in marine invertebrates may change with geography [60], [61], which can contribute to the production of different secondary metabolites. For instance, the geographical variation in the secondary metabolites of the bryozoan *Bugula neritina* is not a result of local adaptation of a single species to a certain habitat, but is rather promoted by the presence or absence of particular bacteria [59]. Given this perspective, future bioprospecting efforts may shift towards microbes. Nevertheless, the challenge will most likely be the successful culture of these symbiotic microorganisms rather than screening for new NP. Isolation may also be an option. However, once isolated from their host it is possible that the symbiotic microorganisms may no longer produce the targeted NP.

### Bioprospecting hotspots: from tropical to polar regions

The geographical analysis concerning sponges and cnidarians found that they were the dominant source of NMNPI since 1990 for most of the latitudinal zones. However, it is interesting to note that echinoderms have recorded the highest number of NMNPI in polar regions. Although sponges are commonly designated as the dominant macroinvertebrates in many Antarctic benthic communities [44], [62], results reveal that echinoderms have been a more important source of NMNP from these regions, particularly the Antarctic. Nonetheless, the number of NMNPI associated with polar regions was lower than that recorded for temperate and tropical regions. A latitudinal hypothesis suggesting an inverse correlation between latitudinal and chemical defense strategies in marine invertebrates has been accomplished based on geographical comparisons on early chemical ecology studies [63]. Following the principle that chemical defense is mainly driven by predation pressure, it was hypothesized that chemical diversity was higher in the tropics than in the poles. Although these extreme regions have not been the focus of most research efforts on the discovery of NMNP, recent studies on Antarctic marine organisms have shown bioactivity levels comparable to those recorded in temperate, and perhaps even tropical, marine environments [44]. Furthermore, recent sampling expeditions by the Antarctic benthic deep-sea biodiversity project in the Southern Ocean revealed extremely high levels of biodiversity across a wide range of taxa [64]. Although results show comparably less NMNPI from polar regions, the number of NMNP per species is similar to that from other regions. This also supports the plausible theory that polar regions have likely potential for the bioprospecting of NMNPI, particularly Antarctica. In this view, the latitudinal differences recorded in this study can be associated with the popularity of temperate and, mostly, tropical environments. Further consideration must be also be given to the remoteness of polar regions and the complexity of logistics to perform research missions on these locations. The accessibility of sampling sites [65] is indeed a possible explanation to the higher number of NMNPI discovered in the northern hemisphere in the past two decades, as this is where most of the world's land area and human population are found. The accessibility to sampling grounds may also explain some results. It is far easier to collect benthic invertebrates in your coastal “backyard”, such as intertidal flats and shallow coral reefs, rather than in remote areas with difficult access, like the deep-sea and polar regions.

Most research on marine invertebrate's chemical ecology has been focused on tropical and temperate environments [44], which was validated by the present study. Results demonstrate the increase in bioprospecting efforts targeting tropical organisms and not, yet, a shift towards untapped regions/habitats. Most of the source organisms of NMNPI discovered since 1990 were associated with BH, which are also more common in tropical regions [18], [66]. Considering the higher biodiversity observed in these regions, one might expect that bioprospecting in the tropics will continue to be the core of NMNP research. Moreover, results indicate a noteworthy increase in bioprospecting in the Pacific Ocean, particularly in Asian countries (e.g. Japan, China and South Korea). In contrast, NMNPI linked to Oceania registered a decrease between the 1990s and the 2000s, which denotes the trends recorded for the Australian and New Caledonian EEZ. This tendency goes against the trends for tropical high-biodiversity regions, as marine biodiversity is remarkably high around Oceania [10], [66]. Three hypotheses can explain such results. The first is associated with the creation of stronger restrictions blocking external researchers from accessing biodiversity within many countries. For instance, in the Queensland state, which encompasses the Great Barrier Reef and contains Australia's highest levels of biodiversity [67], the *Biodiscovery Act 2004* encourages the “development in the State of value added biodiscovery” and “ensures that the State obtains a fair and equitable share in the benefits of the biodiscovery” [68]. Nowadays, all projects related to bioprospecting in the Queensland state have local partners, at least to provide access to the native biota. With such legislation, the sampling of biological material within the Australian EEZ, particularly in Queensland, becomes more difficult for foreign countries that avoid sharing their findings and potential profits with the Australian government. The second possibility is the restriction of trawling activities in many habitats. Benthic trawling is a relatively easy method to collect benthic invertebrates as the biological material is easily captured and brought to the surface. This is often accomplished with cooperation of local fisherman. However, increasing limitations have been applied to trawling activities, particularly when bottom trawling is used in high biodiversity areas [69], which are very attractive for researchers looking for new MNP. Thus, sampling in deeper depths became more difficult, and the access to technology to explore the deep-sea, such as submersibles, is limited. This equipment is very expensive and not affordable by many public or private institutions. The third hypothesis is related to a lower investment on bioprospecting efforts. Funding research addressing the bioprospecting of NMNP can be difficult to obtain due to the strong possibility of failing to discover and/or develop a new drug. Unlike private funding, government funding can offset the high risk factor allowing new national programs to exploit marine biotechnology. New NP discovery have been launched by the governments of Germany, Ireland, Norway and South Korea, but curiously not in the USA within the past decade [10]. These national programs have shown results, principally in the amount of NMNPI associated with the South Korea EEZ (see Figure 4). The observed decrease of NMNPI between decades associated with the USA EEZ might be related with cuts in governmental funds. As regards to Germany, Ireland and Norway, none of these countries recorded a noteworthy number of NMNP discovered from local organisms. Possible reasons for this inconsistency having available funding opportunities are the bioprospecting of other organisms besides marine invertebrates, such as marine bacteria and algae, or the funding of national research teams to bioprospect in foreign countries/EEZ.

The differences observed between country and EEZ information are most likely explained by the collection of organisms overseas by many developed countries. For instance, the NMNPI associated with United Kingdom were often discovered in the EEZ of Bermuda, British Virgin Islands, Falkland and South Georgia EEZ, while for the USA most of the NMNPI were associated with the EEZ of Hawaii and Puerto Rico. Future studies with geographical approaches should present the information regarding EEZ instead of countries. However, the use of EEZ could also be misrepresented, mainly when vast areas are embraced. By comparing results obtained for EEZ and LME it is possible to identify how misleading the use of EEZ can be. For instance EEZ maps (Figure 4) suggest Canada, Russia and USA as important sources of NMNPI since 1990. Nevertheless, most of the NMNPI from the Canada EEZ were associated with the LME Gulf of Alaska, while for the USA EEZ results are biased by the NMNPI associated with the LME Gulf of Mexico. The Russia EEZ outcome is mostly associated with the Sea of Japan and Sea of Okhotsk. Consequently, LME provide a good assessment when EEZ information concerns large areas. Nonetheless, EEZ is still a reasonable approach to analyze information for countries with small EEZ and to contrast the marine biodiversity of each country and its chemical diversity patrimony.

The few EEZ that registered some of the biggest increases between the 1990s and the 2000s were also the ones that recorded most of the NMNPI discovered since 1990, such as the Chinese, South Korean and Taiwanese EEZ). This suggests that in future years these Asian countries will stand out even more as dominant sources of NMNPI. In terms of relevancy as a source of NMNPI, the region surrounding Japan, South Korea and China is to Asia as the Caribbean Sea is to America or the Mediterranean Sea is to Europe. It is also interesting to note that close regions displayed different taxonomical trends in terms of source organisms. Both Bahamas and Puerto Rico belong to the same BH and LME. However, the main sources of NMNPI in the Bahamas were sponges, while in Puerto Rico were cnidarians.

### Conclusion

Even though new technologies provided great advances, particularly in the last two decades, for collecting and studying marine samples in the identification of small amounts of molecules, marine chemical ecology is still several decades behind its terrestrial counterpart [44]. New technologies in analytical spectroscopy have pushed the limits of observation, so that discovery of new molecules requires only a few micrograms — a small portion of the material that was required only 10 years ago [10]. These and further technological developments will enhance the discovery of NMNP, as a small amount of biomass is expected to allow the screening of even more molecules than at the present time. In 1999, marine organisms were already providing larger percentages of bioactive NP than terrestrial organisms [70]. Nevertheless, there is still a large proportion of potential target organisms to be bioprospected [46], particularly marine invertebrates. The findings of this study can help researchers to focus or re-direct their research towards less explored taxonomical groups or geographical regions, to maximize their chances to find NMNP. In contrast, more conservative researchers may want to concentrate their research in taxa and/or regions where high chemical diversity has already been identified.

As the bioprospecting of NMNP becomes increasingly common in coastal regions, mainly because it is easy to sample regions with high biodiversity in shallow habitats, bioprospecting may shift to unexplored regions/habitats, such as the deep-sea. Actually, MNP research in the deep sea has been of increasing interest due to technological advances, with approximately 60% of deep-sea NP reported so far displaying bioactivity [41].

Marine biodiversity conservation has been capturing growing attention by nations worldwide. Nevertheless, to encourage the sustainable use of marine resources one has to protect them. While numerous marine resources have already been severely exploited as they hold great commercial value, several groups of marine invertebrates are still largely undervalued. Nonetheless, most of those marine invertebrates hold a considerable potential towards the development of new products from the sea, namely new drugs. The future of exploration of these organisms may hold great revenues for countries holding the legal rights over the EEZ where bioprospecting efforts take place. Particularly in tropical regions, where most of the bioprospecting of NMNPI has been focused, the protection of marine invertebrate species should be highly encouraged with special attention given to their harvesting and commercialization. It is of paramount importance that the nations possessing these biological resources benefit from potential economic revenues associated with these findings, so that it allows the promotion of social and ecological sustainability.

## References

[1] McGinn AP (1999). Safeguarding the Health of Oceans; Peterson JA, editor..

[2] Ausubel J, Crist DT, Waggoner PE (2010). First Census of Marine Life 2010: Highlights of a decade of discovery..

[3] Paul VJ, Ritson-Williams R (2008). Marine chemical ecology.. Natural Product Reports.

[4] Paul VJ, Puglisi MP (2004). Chemical mediation of interactions among marine organisms.. Natural Product Reports.

[5] Haefner B (2003). Drugs from the deep: marine natural products as drug candidates.. Drug Discovery Today.

[6] Faulkner D (2000). Marine pharmacology.. Antonie van Leeuwenhoek.

[7] Faulkner D (1977). Interesting aspects of marine natural products chemistry.. Tetrahedron.

[8] Chin Y, Balunas M, Chai H (2006). Drug discovery from natural sources.. The AAPS Journal.

[9] Paul VJ, Ritson-Williams R, Sharp K (2011). Marine chemical ecology in benthic environments.. Natural Product Reports.

[10] Molinski TF, Dalisay DS, Lievens SL, Saludes JP (2009). Drug development from marine natural products.. Nature Reviews Drug Discovery.

[11] Wijffels RH (2008). Potential of sponges and microalgae for marine biotechnology.. Trends in Biotechnology.

[12] Harvey A (2000). Strategies for discovering drugs from previously unexplored natural products.. Drug Discovery Today.

[13] Costello MJ, Coll M, Danovaro R, Halpin P, Ojaveer H (2010). A Census of Marine Biodiversity Knowledge, Resources, and Future Challenges.. PLoS One.

[14] Cavanagh R, Gibson C (2007). Overview of the Conservation Status of Cartilaginous Fishes Chondrichthyans) in the Mediterranean Sea.. IUCN.

[15] Sherman K, Sissenwine M, Christensen V, Duda A, Hempel G (2005). A global movement toward an ecosystem approach to management of marine resources.. Marine Ecology Progress Series.

[16] Sala E, Knowlton N (2006). Global marine biodiversity trends.. Annual Review of Environment and Resources.

[17] Spalding MD, Fox HE, Allen GR, Davidson N, Ferdaña ZA (2007). Marine Ecoregions of the Wolrd: A Bioregionalization of coastal and Shelf Areas.. Bioscience Magazine.

[18] Myers N, Mittermeier RA, Mittermeier CG, da Fonseca GAB, Kent J (2000). Biodiversity hotspots for conservation priorities.. Nature.

[19] Blunt J, Copp B, Munro M, Northcote P, Prinsep M (2005). Marine natural products.. Natural Product Reports.

[20] Blunt J, Copp B, Munro M, Northcote P, Prinsep M (2006). Marine natural products.. Natural Product Reports.

[21] Blunt JW, Copp BR, Hu W-P, Munro MHG, Northcote PT (2007). Marine natural products.. Natural Product Reports.

[22] Blunt JW, Copp BR, Hu W-P, Munro MHG, Northcote PT (2008). Marine natural products.. Natural Product Reports.

[23] Blunt JW, Copp BR, Hu W-P, Munro MHG, Northcote PT (2009). Marine natural products.. Natural Product Reports.

[24] Faulkner DJ (1992). Marine natural products.. Natural Product Reports.

[25] Faulkner DJ (1993). Marine natural products.. Natural Product Reports.

[26] Faulkner DJ (1994). Marine natural products.. Natural Product Reports.

[27] Faulkner DJ (1995). Marine natural products.. Natural Product Reports.

[28] Faulkner DJ (1996). Marine natural products.. Natural Product Reports.

[29] Faulkner DJ (1997). Marine natural products.. Natural Product Reports.

[30] Faulkner D (1998). Marine natural products.. Natural Product Reports.

[31] Faulkner DJ (1999). Marine natural products.. Natural Product Reports.

[32] Faulkner DJ (2000). Marine natural products.. Natural Product Reports.

[33] Faulkner DJ (2001). Marine natural products.. Natural Product Reports.

[34] Faulkner DJ (2002). Marine natural products.. Natural Products Reports.

[35] Blunt J, Copp B, Munro M, Northcote P, Prinsep M (2003). Marine natural products.. Natural Product Reports.

[36] Blunt J, Copp B, Munro M, Northcote P, Prinsep M (2004). Marine natural products.. Natural Product Reports.

[37] Blunt JW, Copp BR, Munro MHG, Northcote PT, Prinsep MR (2010). Marine natural products.. Natural Product Reports.

[38] Blunt JW, Copp BR, Munro MHG, Northcote PT, Prinsep MR (2011). Marine natural products.. Natural Product Reports.

[39] Appeltans W, Bouchet P, Boxshall GA, Fauchald K, Gordon DP (2011). World Register of Marine Species.. http://www.marinespecies.org.

[40] Ianora A, Boersma M, Casotti R, Fontana A (2006). New trends in marine chemical ecology.. Estuaries and Coasts.

[41] Skropeta D (2008). Deep-sea natural products.. Natural Product Reports.

[42] Bergmann W, Burke D (1955). Contributions to the study of marine products. XXXIX. The nucleosides of sponges.. III.1 Spongothymidine and spongouridine2 Journal of Organic Chemistry.

[43] Blunt JW, Munro MHG (2008). Dictionary of Marine Natural Products; Blunt JW, Munro MHG, editors..

[44] Avila C, Taboada S, Núñez-Pons L (2008). Antarctic marine chemical ecology: what is next?. Marine Ecology.

[45] Faulkner DJ (2000). Highlights of marine natural products chemistry (1972–1999).. Natural Product Reports.

[46] Marris E (2006). Drugs from the deep.. Nature.

[47] Harvey A (2008). Natural products in drug discovery.. Drug Discovery Today.

[48] Laport M, Santos O, Muricy G (2009). Marine sponges: potential sources of new antimicrobial drugs.. Current Pharmaceutical Biotechnology.

[49] Su J-H, Lin Y-F, Yeh H-C, Wang W-H, Fan T-Y (2009). Oxygenated cembranoids from the cultured and wild-type soft corals *Sinularia flexibilis*.. Chemical & Pharmaceutical Bulletin.

[50] Yu S, Deng Z, Ofwegen L, Proksch P, Lin W (2006). 5,8-Epidixysterols and related derivatives from chinese soft coral *Sinularia flexibilis*.. Steroids.

[51] Lin Y-S, Chen C-H, Liaw C-C, Chen Y-C, Kuo Y-H (2009). Cembrane diterpenoids from the taiwanese soft coral *Sinularia flexibilis*.. Tetrahedron.

[52] Duh C-Y, Wang S-K, Tseng H-K, Sheu J-H (1998). A novel cytotoxic biscembranoid from the formosan soft coral *Sinularia flexibilis*.. Tetrahedron Letters.

[53] Ramesh P, Reddy N, Rao T, Venkateswarlu Y (1999). New oxygenated africanenes from the soft coral *Sinularia dessecta*.. Journal of Natural Products.

[54] Wen T, Ding Y, Deng Z, Ofwegen L, Proksch P (2008). Sinulaflexiolides A-K, cembrande-type diterpenoids from the chinese soft coral *Sinularia flexibilis*.. Journal of Natural Products.

[55] McFadden CS, Sanchez JA, France SC (2010). Molecular Phylogenetic Insights into the Evolution of Octocorallia: A Review.. Integrative and Comparative Biology.

[56] Davidson S, Haygood M (1999). Identification of sibling species of the bryozoan Bugula neritina that produce different anticancer bryostatins and harbor distinct strains of the bacterial symbiont "Candidatus Endobugula sertula".. The Biological Bulletin.

[57] Bickford D, Lohman DJ, Sodhi NS, Ng PKL, Meier R (2007). Cryptic species as a window on diversity and conservation.. Trends in Ecology & Evolution.

[58] Duckworth A (2009). Farming Sponges to Supply Bioactive Metabolites and Bath Sponges: A Review.. Marine Biotechnology.

[59] McGovern T, Hellberg M (2003). Cryptic species, cryptic endosymbionts, and geographical variation in chemical defences in the bryozoan Bugula neritina.. Molecular Ecology.

[60] Hentschel U, Hopke J, Horn M, Friedrich AB, Wagner M (2002). Molecular evidence for a uniform microbial community in sponges from different oceans.. Applied and Environmental Microbiology.

[61] Hay M, Fenical W (1996). Chemical ecology and marine biodiversity: insights and products from the sea.. Oceanography.

[62] Dayton P, Robilliard G, Paine R (1974). Biological accommodation in the benthic community at McMurdo Sound, Antarctica.. Ecological Monographs.

[63] Bakus G (1974). Toxicity in holothurians: a geographical pattern.. Biotropica.

[64] Brandt A, Debroyer C, Gooday A, Hilbig B, Thomson M (2004). Introduction to ANDEEP (ANtarctic benthic DEEP-sea biodiversity: colonization history and recent community patterns)—a tribute to Howard L. Sanders.. Deep Sea Research Part II: Topical Studies in Oceanography.

[65] Tan G, Gyllenhaal C, Soejarto DD (2006). Biodiversity as a source of anticancer drugs.. Current drug targets.

[66] Roberts CM (2002). Marine Biodiversity Hotspots and Conservation Priorities for Tropical Reefs.. Science.

[67] Hooper J (2007). Maximizing benefits from ‘biodiscovery’: a Coastal State resource providers perspective.. United Nations Informal Consultative Process on Oceans and Law of the Sea.

[68] Spranger T (2005). Queensland's Biodiversity Act 2004.. Journal of International Biotechnology Law.

[69] Gianni M (2004). High seas bottom trawl fisheries and their impacts on the biodiversity of vulnerable deep-sea ecosystems: options for international action..

[70] Munro M, Blunt J, Dumdei E, Hickford S, Lill R (1999). The discovery and development of marine compounds with pharmaceutical potential.. Journal of Biotechnology.

